# A Comprehensive Review on Lane Marking Detection Using Deep Neural Networks

**DOI:** 10.3390/s22197682

**Published:** 2022-10-10

**Authors:** Abdullah Al Mamun, Em Poh Ping, Jakir Hossen, Anik Tahabilder, Busrat Jahan

**Affiliations:** 1 Faculty of Engineering and Technology, Multimedia University, Melaka 75450, Malaysia; 2 Department of Computer Science, Wayne State University, Detroit, MI 48202, USA; 3 Department of Computer Science and Engineering, Feni University, Feni 3900, Bangladesh

**Keywords:** ADAS, deep neural network (DNN), DBSCAN, object detection, segmentation

## Abstract

Lane marking recognition is one of the most crucial features for automotive vehicles as it is one of the most fundamental requirements of all the autonomy features of Advanced Driver Assistance Systems (ADAS). Researchers have recently made promising improvements in the application of Lane Marking Detection (LMD). This research article has taken the initiative to review lane marking detection, mainly using deep learning techniques. This paper initially discusses the introduction of lane marking detection approaches using deep neural networks and conventional techniques. Lane marking detection frameworks can be categorized into single-stage and two-stage architectures. This paper elaborates on the network’s architecture and the loss function for improving the performance based on the categories. The network’s architecture is divided into object detection, classification, and segmentation, and each is discussed, including their contributions and limitations. There is also a brief indication of the simplification and optimization of the network for simplifying the architecture. Additionally, comparative performance results with a visualization of the final output of five existing techniques is elaborated. Finally, this review is concluded by pointing to particular challenges in lane marking detection, such as generalization problems and computational complexity. There is also a brief future direction for solving the issues, for instance, efficient neural network, Meta, and unsupervised learning.

## 1. Introduction

Autonomous driving has become a hotspot research topic as the intelligent transport system and environmental perception improves daily. LMD is one of the significant parts of the environmental perception system, where many efforts have been made in the previous decade. Nevertheless, developing an efficient lane detection framework under different environmental circumstances is a highly challenging task because it has many dependencies that may influence the framework’s final output.

Various preprocessing techniques have a significant role in lane marking detection systems, mostly dependent on heuristic features. Distinct types of filters such as Finite Impulse Response (FIR) [1], Gaussian [2], and mean and median [3] are used to remove the noise from the input dataset. Duan et al. [4] introduced threshold segmentation to deal with the variation in illumination. Additionally, PLSF [5] and Otsu [6] are also applied for the same region. There are different Regions of Interest (ROI) that are examined to avoid redundancy, such as vanishing point-based ROI [7], adaptive ROI [8], and Fixed-size ROI [9]. An essential preprocessing tool to enhance the quality of lane marks is colour conversion, such as the RGB to HSV colour model.

There are many algorithms applied to extract lane features, especially for straight lanes, for instance, Hough [10], Canny [8], Sobel [9], and FIR filter [11]. Catmull–Rom spline [12], clothoid curve [13], parabolic [14], and cubic B-spline [2] are applied for curved lanes. A few other techniques are used under complex conditions, such as image enhancement [15] and wavelet analysis [16].

DNN (Deep Neural Network) has become one of the most promising computer vision techniques since AlexNet won the ILSVRC challenge in 2012. These deep learning techniques have shown promising performances in various fields of research. Recently, various efficient deep learning approaches have been examined for lane marking detection. From the beginning, Convolution Neuron Network (CNN) [17,18] to the GAN-based method [19] and segmentation process [20] have obtained efficient results on LMD. Additionally, DAGMapper [21] and attention map [22] have been applied to understand the structural features of the lanes. Though these techniques have obtained auspicious results, LMD is still challenging for its lack of generalization capability. For instance, a trained model in a particular scenario, such as daytime, may obtain poor results in other environmental scenarios, such as nighttime.

This article provides an efficient, comprehensive review of LMD using different deep neural networks. The manuscript provides a review of the complete process of lane marking detection (LMD) using deep learning techniques, considering the sequential process. It provides a clear indication of preprocessing and post-processing approaches for lane marking detection. In addition, the manuscript provides an optimization process to improve the algorithm as well as to remove the post-processing steps. The loss function is an important part of LMD, and it is categorically discussed, broken into classification, regression, and adversarial training to make it these categories easy to understand. Deep learning algorithms for LMD are explained in three major categories: object detection, classification, and segmentation of lanes, to cover all aspects of this field with proper objectives, limitations, suggested improvements, and structures. The summary table (Table 1 and Table 2) provides the deep learning algorithms for LMD with achievements, results and constraints to indicate their goals and barriers. More importantly, a discussion shows a comparative result with outcome figures by training and testing the models with the Tusimple dataset. Finally, a future direction is provided to give a probable option for improving the LMD techniques. The remaining sections of the review article are organized as follows: Section 2 outlines distinct deep learning techniques (including preprocessing, loss function, cluttering, and preprocessing) for LMD. Section 3 describes the comparative result of some experimental results. Finally, Section 4 gives the conclusion and future thoughts on LMD techniques.

## 2. LMD Using DNN

The existing lane marking detection approaches can be classified into two major categories: single-stage and two-stage [23]. The initial segment of the two-stage frameworks extracts the heuristic recognition and deep learning-based lane features. In contrast, the second segment refers to the post-processing steps, which may include fitting, clustering, or interfacing. However, the single-stage lane detection approach provides final results directly from the input stage, including post-processing and cluster results. The LMD using the deep neural network has been discussed from four perspectives: preprocessing, network architecture, network loss functions, and post-processing.

### 2.1. Pre-Processing

ROI cropping is applied to remove the irrelevant information from the input dataset in the traditional and initial parts of the deep learning approaches. Consequently, it reduces the computational complexity and increases the running speed of the framework. As the lane markings are visualized on the lower part of the image frames, the clipped portion refers to the frames’ upper or sky part. Thus, it reduces the computational complexity by around 30% [23].

**Figure 1:**
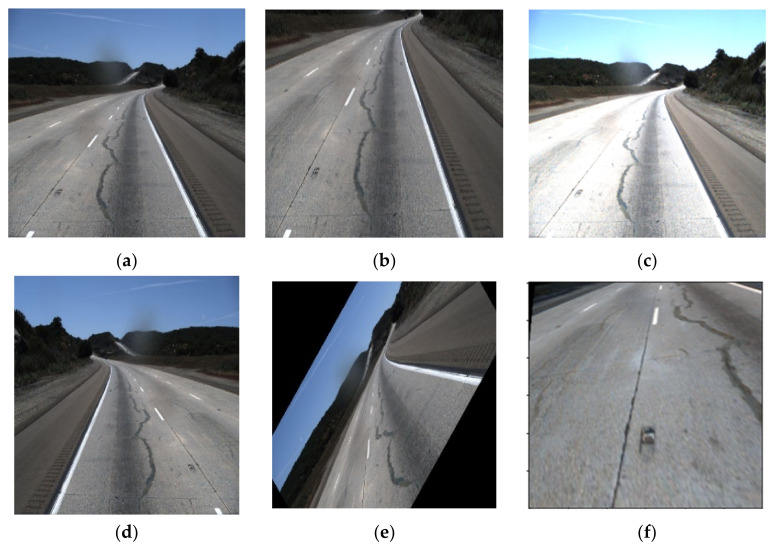
Different pre-processing technique (**a**) original, (**b**) cropped, (**c**) brighten (**d**) mirrored, (**e**) rotating and (**f**) perspective.

Some advanced techniques, such as meta-learning, can be examined to ameliorate the generalization of the CNN method. It can also be improved by diversifying the training dataset. The augmentation technique has a significant role in diversifying and increasing the number of data in the image dataset. In this process, data can be cropped, rotated, brightened, and mirrored to assort the training dataset shown in Figure 1 as a reference.

### 2.2. Network Architecture of LMD

There are many strategies to detect the LMD using a deep learning network, though these strategies can be categorized based on defining the LMD task. Therefore, these techniques can be classified as object detection, classification, and segmentation of lanes. Every feature point on lane segments is labeled, and detects the lanes as an object by the regression coordinates. In comparison, lane position is determined by combining the prior information in the classification techniques. On the contrary, background and lane pixels are labelled as distinct classes and detect the lane through semantic or instant segmentation. However, some LMD techniques are also satisfied with multiple purposes along with detecting lane marks, such as road marking detection, road type classification, and drivable area detection. Initially, architectural information can be managed from the primary convolution network, such as ResNet, VGG, and FCN.

#### 2.2.1. The Initial Network Architecture of LMD

**Figure 2:**
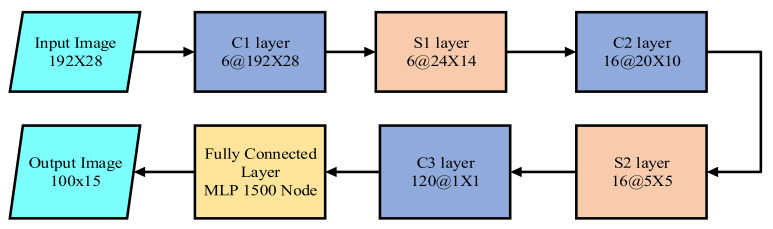
The architecture of CNN based lane marking detection technique.

CNN was first introduced to extract the lane feature in LMD by Kim et al. [17]. Additionally, random sample consensus (RANSAC) was used to group the identical architecture of the lane locations. The CNN architecture, shown in Figure 2, consists of three convolution layers, two subsampling layers, and three fully connected layers (FCL). The input dataset was converted into 192 × 28 after the ROI and edge detection. The last FCL provided the predicted output of 100 × 15.

Though it has improved LMD compared to the traditional methods, it also has some research limitations. The approach requires complex data processing unit and has a complex architecture of eight layers. Therefore, other researchers have developed other improved deep neural networks to overcome the existing limitations.

#### 2.2.2. Lane Detection Based on Object Detection

Various types of visual detection systems are available for the autonomous driving system, such as road marking detection, vehicle detection, and, most importantly, lane marking detection. Sermanet et al. [24] introduced the overfeat technique, emphasizing the importance of a multi-supervised training approach, which simultaneously improved performance due to location, detection, and classification. Two key points typically focus on object detection, such as predicting the object and position of the object on the image.

Huval et al. [25] introduced empirical evaluation of the deep learning (EELane) technique with an overfeat detector to detect the highway’s lane markings. This research aims to apply six regressions to predict the lanes. The initial four regression dimensions indicate the finishing aspects of the line under the segmented lane boundary. The reaming regression dimension conceding the camera suggests the more profound finishing points. The geometrical information from CNN has been applied for many purposes, such as edge detection and inpainting, to assist the main task. The reader can go through it for a detailed understanding [26].

**Figure 3:**
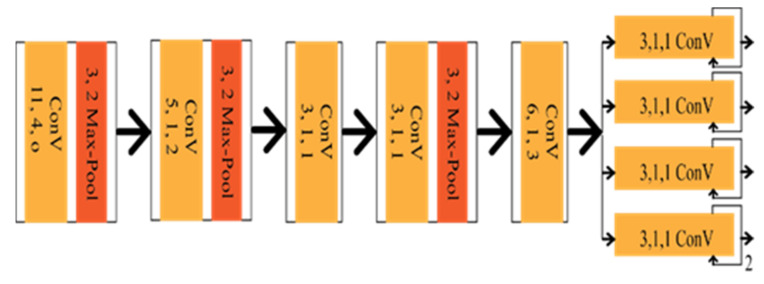
Schematic diagram of VPGNet.

Seokju et al. [27] introduced VPGNet based on VPD, also a geometric estimation method of CNN. It is a modified version of the vanishing point tracking method, composed of four segments. The Vanishing point can guide road marking recognition and lane detection, which was the main contribution of the VPGNet. VPGNet has some post-processing framework for lane regression and clustering, increasing computational complexity. The architecture of the network is shown in Figure 3.

**Figure 4:**
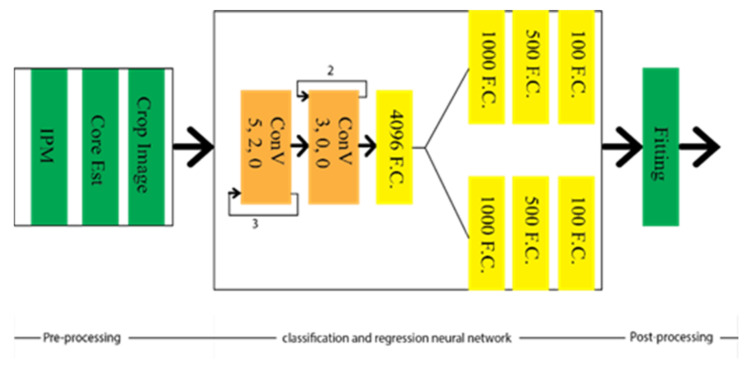
Schematic diagram of spatial and temporal based LMD technique.

EELane and VPGNet showed the effectiveness of the multi-branch techniques where lane detection can be guided from prior knowledge by sharing different tasks into contiguous representations. Huang et al. [28] combined the spatial and temporal data in the CNN framework to detect the lane markings by selecting the lane boundaries. Therefore, the computational time is reduced, allowing it to be run more effectively in the automated driving car under intricate weather and traffic schemes in real-time. With this aim in mind, lane location estimation is obtained by evaluating Inverse Perspective Transformation (IPM) from the overhead view of the images using spatial and temporal relevancy of lanes. The images are cropped into relevant sub-image, carrying out the local lanes’ boundary information. Sequentially, the CNN framework is applied to detect the actual location and boundary of the lanes. The final structure is optimized to reduce the computational complexity by selecting the adjacent lanes based on the lane change for searching for the lanes’ actual position. The architecture of the network is depicted in Figure 4. The study of spatial and temporal relevancy of lanes made it different from EELane and VPGNet, whereas IM’s implementation created the condition of its robustness. However, this makes up for low illumination conditions, such as at night and rainy conditions [28].

#### 2.2.3. Lane Detection Based on the Classification

**Figure 5:**
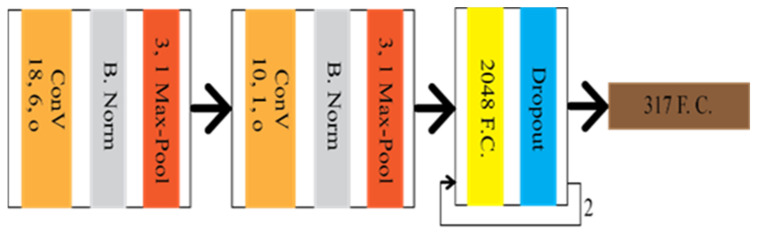
Schematic diagram of DeepLane.

Image classification refers to the discrimination process of objects available in the input image frame. However, the location of the lane can not be tracked through this process. Therefore, some modification is required in the classification technique to track the lane’s location. Let us consider the amendment on the classification is y = f(x,pm(p)), where f(x) is the CNN mapping function, and pm(p) is the prior knowledge depending on the lane location. Gurghian et al. [18] have come up with DeepLane depending on the same idea, which network architecture is shown in Figure 5. DeepLane received the training dataset, which was created from the image frames of the downward camera. It was classified into 317 classes, among which, 316 were for the probable lane position and reaming one was for missing lanes. A softmax function was applied to the last fully connected layer to achieve the probability distribution. The lane position was estimated *E_i_* through the following equation:

$$E_{i}=argmax\left(y_{i}\right),\;0\leq i\leq 316,\;\mathrm{where},\;y_{i}=y_{o},y_{1},\ldots \ldots .,y_{316}$$

Though DeepLane has achieved a better result than a complex network [17], the prior fixing of the lane position has limited its robustness. In addition, the classification techniques do not fit with lane marking detection, as it is associated with the high-level task. As discussed earlier, the regression of the lane coordinate as an object detection process is also a better possible way to detect lane marking detection.

#### 2.2.4. Lane Detection Based on the Segmentation

Segmentation approaches such as [29,30,31] can be the best option for lane marking detection, as mentioned by Shriyash et al. [32]. These approaches strictly emphasize per-pixel classification rather than focusing on particular shapes. Lane detection based on the segmentation framework achieved more efficient results, except for the concern of the above limitation. This problem is solved by many strategies, such as the strategy proposed by Chiu et al. [33], which referred to the lane marking detection system as an image segmentation problem. However, the conventional segmentation approaches did not last long.

- End-to-End Segmentation Approach

Due to the previous reason, the researcher started to apply end-to-end segmentation approaches for lane marking detection. The network can carry more features according to the larger size of the convolution kernel. Zhang et al. introduced a GCN [34] algorithm to detect particular lane areas. A lane departure system based on Mask-RCNN [35] is proposed by Riera Luis et al. to detect the lane marks and an additional Kalman filter to track the lanes. Shriyash et al. [36] proposed a CNN architecture that consists of ten neuron layers to detect the lanes in real time. Different types of lanes also have a notable contribution to more comprehensive recognition detection. The modified ERFNet architecture was designed by Fabio et al. [37] to classify the road lanes and identify the drivable area.

Semantic Segmentation through DCNN may have some deficiencies, as it has no learnable pooling parameters. For instance, there is no learnable parameter in max/min pooling or un-sampling layers. Therefore, there is an extreme possibility of losing many features when attempting to recognize a large-perspective field. Kontun et al. [38] introduced dilated convolution to resolve this issue, which can be studied more in [39]. Though this framework had significant advantages, the effective design of CNN architecture emphasizing dilated convolution has become a new issue.

**Figure 6:**
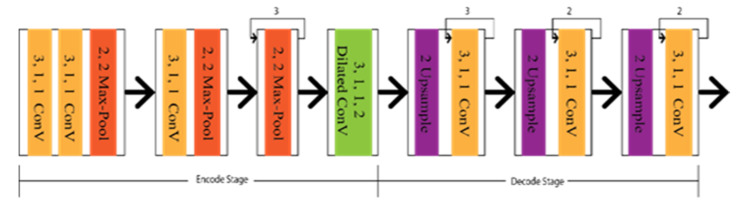
Schematic diagram of Deep Convolution Neural Network based on the lane markings detector (LMD).

Chen et al. proposed a Deep Convolution Neural Network based on the lane markings detector (LMD), aiming to have the optimal CNN architecture design with dilated convolution [40]. The lane markings detector, similar to ResNet [41] and VGG [42], is used as an encoder to classify, and DeconvNet [43], U-Net [44], and FCN [45] are used as a decoder to create feature maps. Additionally, dilated convolutions were embedded in the encode–decode section of the architecture shown in Figure 6. Lo et al. [40] introduced a CNN architecture based on DDB (Digressive Dilation Block) and FSS (Feature Size Selection), considering the spatial and downsampling operation, which was also embedded with dilation convolution [46].

Long-range information in lane marking detection is another concern. Wang et al. [47] designed a non-local operation depending on a non-local framework [48]. The model could extract the long-distance or range information, as long-distance information is also one of a lane’s properties. Li et al. [49] proposed Instance batch normalization and Attention Network (IANet) to emphasize the model for considering a particular lane region. It is more appropriate for two-class segmentation scenarios, according to the experimental result.

**Figure 7:**
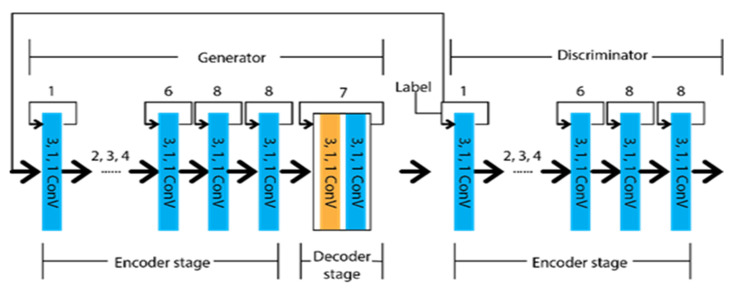
Schematic diagram of EL-GAN.

Considering efficient classification by focusing on pixels rather than shape, Jan et al. [50] came up with an adversarial network known as generative adversarial networks (GAN). It has a generator to create the synthetic data and a discriminator to differentiate the real data from the generator’s output data. The initial concept for the GAN was to predict data closely approximate to the real data. The recent concept tells us to differentiate accurately to determine whether the input is generated or real. A reader can go through [51,52,53] for further information about the GAN. Ghafoorian et al. [19] designed Embedding loss GAN (EL-GAN) based on the GNN concept. The framework is divided into two segments, as generator and discriminator. The schematic diagram of the EL-GAN framework is shown in Figure 7. U-Net’s unique algorithm is applied for the generator to train the input, and Tiramisu DenseNet [54] is used for detecting the lane markings. This process is continued up to the level of convergence. In the case of the discriminator, DenseNet [55,56] is used with the fully connected Generative Adversarial Network classification [57].

The framework generator is trained by adversarial embedding and Adam optimizer, whereas the discriminator is trained by stochastic gradient descent and ordinary cross-entropy. Embedding loss can be considered perceptual loss [58], whereas EL-GAN combines perceptual loss and CGAN.

- Segmentation based on multitask

**Figure 8:**
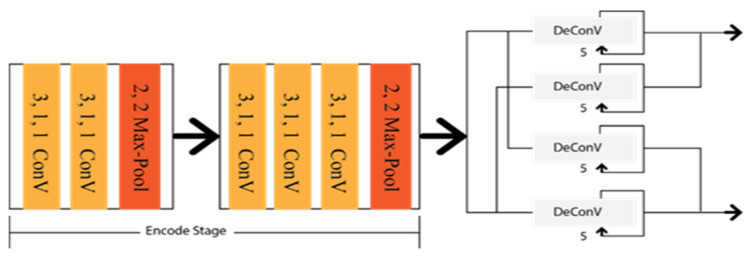
Schematic diagram of GLCNet.

Geometrical features of roads also have an important role in lane marking detection, which have better performance results than VPGNet. Zhang et al. [59] proposed Geometric Constrained Network (GLCNet), which has multitasked to interlink the lane boundary and lane segmentation sub-structure. The architecture of GLCNet [59] is shown in Figure 8, which indicates that every decode section has a link with the encode section to transfer corresponding features into two distinct tasks. Therefore, the information from the decode sections can be redounded reciprocally. This multitask strategy opened the gate for the researchers to develop a framework for the link between lane boundary and lane area. Considering the same idea as GCLNet, John et al. [60] designed PSINet for multiple detection purposes, such as road scene labels, lane marks, and free space on the road.

**Figure 9:**
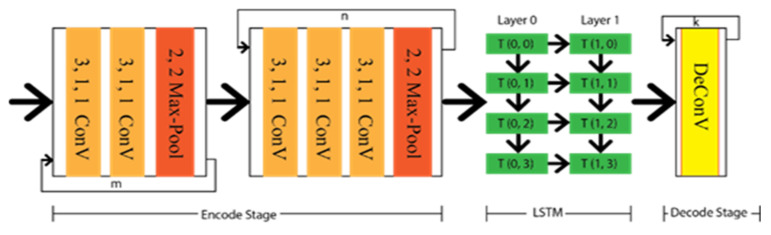
Schematic diagram of CNN-LSTM.

In addition to the geometric or special feature, temporal correlation might have a significant effect where a lane can not be detected due to the linear structure of the captured video. As Long short-term memory (LSTM) has memory capture capability, the lane can be extracted from the previous frame by this LSTM approach. Hence, Qin et al. [61] proposed a CNN-LSTM method that includes two LSTM layers between the encode–decode stage. The major achievement of this method is that it has obtained ameliorate performance results under different occlusion scenarios. The architecture of the CNN-LSTM method is depicted in Figure 9, which indicates the temporal information transfer between the encode–decode stage through LSTM.

#### 2.2.5. Simplification of the Post-Processing Step

Without considering the optimization by the post-processing step, the described frameworks extracted lane features more efficiently. It is very challenging to differentiate the lane features from the output, excluding the post-processing approach. Effective strategies are more important than particular network architecture to discover the optimal result. This sub-section focuses on these strategies, rather than a deep neural network (DNN) architecture, on lane marking detection.

There are two types of algorithmic output possible for lane marking detection using DNN, such as lane points and lane lines. Hence, the possibilty is raised to utilize different lane features, excluding post-processing steps. There might be three possible solutions to overcome the particular constraint: semantic segmentation by labelling each line as separate classes, instance segmentation by referring to every lane as a different instance, and multi-branch CNN structure by detecting every lane line through the individual branch.

**Figure 10:**
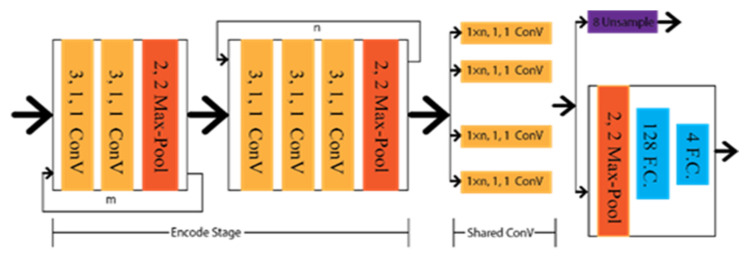
Schematic diagram of SCCN.

Xingang et al. [20] applied a Spatial Convolution Neural Network (SCNN) to detect the lanes under occlusion scenarios as multi-class semantic segmentation. SCNN framework is based on the LargeFOV layout [62], and the weight of the initial thirteen convolution layers is taken from VGG16 [42]. To predict the lanes precisely, it generates pixel-wise probability maps for training the network. Consequently, it applies a CNN to differentiate the lane markings on its own. Finally, the probability maps are sent to the system to predict the lane markings of different classes. The architecture of the SCCN is shown in Figure 10, where various branches were designed to predict other lane classes.

**Figure 11:**
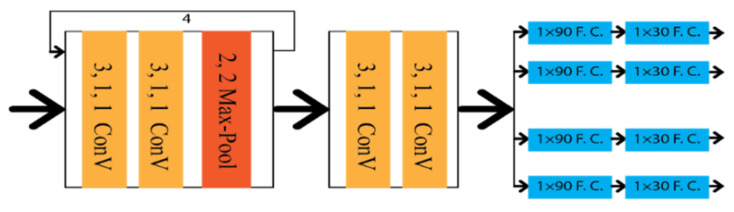
Schematic diagram of CooNet.

Shriyash et al. [32] proposed Coordinate Network (CooNet) as a lane point regression approach. It is a multi-branch neural network shown in Figure 11, where lanes are predicted in their perspective branches. However, this network has no clustering process as the network directly provides the lane output through the coordinate regression.

**Figure 12:**
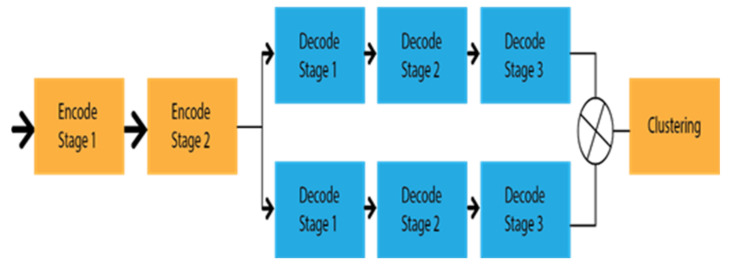
Schematic diagram of Lanenet.

To detect multi lanes with changes from the lanes, Davy et al. [63] introduced an end-to-end lane detection approach by applying the LaneNet deep learning method based on the encoding–decoding procedure E-Net [64], as shown in Figure 12. It takes the shared encodes from the input images and finds the embedding binary segmentation for each pixel for creating the cluster together. All pixels can associate with the neighbourhood pixels. It utilized the H-Net to collect the ideal information about the perspective transformation by imposing a relevant condition on the input image. The research aimed to take the challenge on lane changes, unlike the bird’s eye view. Additionally, this approach has no limitation on the number of lanes, whereas CooNet and SCNN can only detect up to four lanes.

#### 2.2.6. Optimization Approaches

There is always a scope to improve the existing performance in the perspective research field. Still, there is a particular opportunity to optimize the lane marking detection process, as some research limitations exist for that particular application. The new question is, how can one researcher design a framework utilizing a trained model? The answer has come from the transfer learning technique and the knowledge distillation approach.

The dataset for transfer learning can be categorized into the target and source datasets. The target dataset relates to the task directly, and the source dataset indicates an additional dataset for the task. Fine-tuning becomes the major challenge due to the presence of both datasets in transfer learning. Hinton et al. [65] proposed a solution by introducing knowledge distillation, where the teacher network is used to guide the student network, which contains small parameters. It improves the performance of the student network. A few other researchers [66,67] enriched the knowledge distillation into attention distillation. This idea significantly improved lane marking detection when Kim et al. [68] designed Transfer Learning for Ego Lane detection (TLELane). In the TLELane architecture, two transfer learning stages differentiate the general scene from the road scene and capture the target lane to the left to right ego lane from that particular general scene. The attention map extracts high contextual features from different perspective layers in the trained lane marking detection segmentation-based network. These extracted features hold information regarding the rough outline and lane location. Thus, it is a promising way to replicate attention maps for the deeper block by utilizing the initial block. Apart from attention dilation, Hou et al. [22] introduced a self-learning distillation known as the self-attention distillation framework.

There is also a way to remove the post-processing steps, including clustering. Thus, the CNN needs to carry both the predicted lane and parametric description of each lane. Ze et al. [69] designed a combinational neural network with CNN and LSTM named Real-time Lane Network (RLaneNet). LSTM can face an uncertain number of lanes and also has a decoder to retain the parameter information of each lane. According to the mathematical assumption, a lane can be drawn from three corresponding coordinate points and a quadratic function. Based on this assumption, RLaneNet predicted three corresponding coordinate points of the lane that intersect the lane line with three horizontal lines. In contrast, Differentiable Least-squares Fitting Network (DLFNet) [70] lane curvature parameters are estimated by the weight of the least squares. These weights are captured from a deep neural network, and a geometrical loss function minimizes the area between ground truth and lane. The least-squares fitting can be defined as the following equation:

NXα = NY, where X and Y are coordinate matrices, N is the weighted pixel map, and α is the best-fitting curve parameter.

#### 2.2.7. Loss Function in LMD Networks

The measurement of loss of the deep neural network is another key factor in making predicted data consistent with the ground truth data. It also ensures the optimization of the neural network. Different loss functions such as classification, regression, and adversarial training have different tasks in the network discussed in this section. The loss function for classification techniques is discussed below.

Cross-Entropy (CE), *L*_1,_ and *L*_2_ loss have been used most in the case of pixel-level and lane line classification. The equation of *L*_1_ and *L*_2_ can be derived as follows:

(1) $$L_{1}\left(\hat{y},x\right)=\frac{1}{hwm}\sum_{r,s,t}\left|{\hat{y}}_{r,s,t}-x_{r,s,t}\right|$$

(2) $$L_{2}\left(\hat{y},x\right)=\frac{1}{hwm}\sum_{r,s,t}{\left({\hat{y}}_{r,s,t}-x_{r,s,t}\right)}^{2}$$

where *h* refers to height, *w* refers to weight, and *m* refers to the number of channels. Additionally, *x* and $\hat{y}$ represent the corresponding input and output.

The CE loss function can adopt the interclass competition mechanism, as indicated in Equation (3).

(3) $$L_{ce}=-\sum_{n}^{c}n_{i}\log\left(m_{i}\right)$$

where *m_i_* is predicted probability and n_1_ is the class. For *C* = 2, the CE loss equation becomes Equation (4).

(4) $$L_{bce}=-\sum_{n=1}^{c\prime =2}-n_{i}\log\left(m_{i}\right)=-n_{1}\log\left(m_{1}\right)-\left(1-n_{1}\right)\log\left(1-m_{1}\right)$$

Weighed CE loss function, as shown by Equation (5), is used when unbalance exists in the sample data. For instance, there is a big unbalance ratio between background and lane line areas. Zou et al. settled the weight for the background and lane line by 1.0.

(5) $$L_{wce}=-\sum_{i}^{c}w_{i}n_{i}\log\left(m_{i}\right)$$

In the case of significant errors, *L*_1_ and *L*_2_ are more sentient compared to small errors. Let us consider the simplified loss function of *L*_2_ as:

(6) $$J=\frac{1}{2}{\left(y_{i}-{\hat{y}}_{i}\right)}^{2}\;\mathrm{where},\;{\hat{y}}_{i}=\left(Wx_{i}+b\right)$$

(7) $$\frac{dJ}{dW}=\left(y_{i}-{\hat{y}}_{i}\right)\sigma^{\prime }\left(Wx_{i}+b\right)x_{i}$$

From the above partial derivative, if the value of $\sigma \left(Wx_{i}+b\right)$ becomes near 0 or 1, the derivative will also become 0, indicating an initial slow divergence. However, the derivative CE, as in Equation (8), does not depend on another multiplication term to have the possibility of bringing 0. Therefore, the CE loss function is more applicable in lane marking detection applications, mostly on semantic segmentation.

(8) $$\frac{dL_{ce}}{dW}=\left[\sigma \left(m_{i}\right)-y_{i}\right]\cdot x_{i}$$

However, there is a possibility of scattering the learned feature, since the CE loss function only focused on the correct label, ignoring the difference between the incorrect label. The different solutions were dependent on the perspective function. Authors have used A-Softmax [71] and L-Softmax [72] functions, considering the perspective activation function. Contrastingly, Zhang et al. [59] proposed IoU loss, considering the perspective loss function, which indicates the relationship between ground truth and predicted probability. The loss function for regression techniques is discussed below in detail.

Coordinate and grid regression is based on the distance measurement used in [31,35,73]. Coordinate regression can be defined as Equation (9):

(9) $$L_{\mathrm{colour}}=\sum_{i=1}^{15}\left|xc_{i}-xg_{i}\right|+\sum_{i=1}^{15}\left|yc_{i}-yg_{i}\right|$$

where $xg_{i}$ and $yg_{i}$ represent the coordinate of the ground truth, and $xc_{i}$ and $yc_{i}$ represent the corresponding predicted coordinate. At the same time, the grid regression can be expressed as Equation (10):

(10) $$\begin{array}{c} L=\lambda_{\mathrm{coord}}\sum_{n=0}^{R^{2}}\sum_{m=0}^{t}1_{nm}^{obj}\left[{\left(x_{n}-{\hat{x}}_{n}\right)}^{2}+{\left(y_{n}-{\hat{y}}_{n}\right)}^{2}\right]\\ \;+\lambda_{\mathrm{coord}}\sum_{n=0}^{R^{2}}\sum_{m=0}^{t}1_{nm}^{obj}\left[{\left(\sqrt{w_{n}}-\sqrt{{\hat{w}}_{n}}\right)}^{2}+{\left(\sqrt{h_{n}}-\sqrt{{\hat{h}}_{n}}\right)}^{2}\right] \end{array}$$

where, $x_{i}$, $y_{i}$ are the centre coordinates, $w_{i}$, $h_{i}$ are the weight and height of the ground truth, and ${\hat{x}}_{i},{\hat{y}}_{i},{\hat{w}}_{i},{\hat{h}}_{i}$ comprise the corresponding prediction.

The loss function for adversarial techniques is discussed below in detail.

Generative adversarial networks (GAN) have created a different computer vision task with a generator to create the synthetic data and a discriminator to differentiate the real data from the generator’s output data. GAN’s loss function is defined as Equations (11)–(13), which is a modified version of CE.

(11) $$\underset{c}{min}\underset{B}{max}V(B,C)=E_{x∼P\left(x\right)}\left[\log B\left(x\right)\right]+E_{y∼P_{y}\left(y\right)}\left[\log\left(1-B\left(C\left(y\right)\right)\right)\right]$$

(12) $$\underset{B}{max}V(B,C)=E_{x∼\mathrm{Pdata}\left(x\right)}\left[\log B\left(x\right)\right]+E_{y∼P_{y}\left(y\right)}\left[\log\left(1-B\left(C\left(y\right)\right)\right)\right]$$

(13) $$\underset{B}{max}V(B,C)=E_{x∼\mathrm{Pdata}\left(x\right)}\left[\log B\left(x\right)\right]+E_{y∼P_{y}\left(y\right)}\left[\log\left(1-B\left(C\left(y\right)\right)\right)\right]$$

Ghafoorian et al. [19] used various types of losses in the EL-GAN method, such as Cross-Entropy Loss $L_{ce}$, $L_{2}$ loss and adversarial loss *L_ad_* are indicated in Equations (14)–(16), respectively.

(14) $$\begin{array}{c} L_{ft}=L_{jt}\left(G\left(x;\theta_{gen}\right),y\right)=L_{ce}\left(G\left(x;\theta_{gen}\right),y\right)\\ L_{ce}\left(\dot{y},y\right)=\frac{1}{wh}\sum_{i}^{wh}\sum_{j}^{c}y_{i,j}\ln\left({\dot{y}}_{i,j}\right) \end{array}$$

(15) $$L_{2}\left(\dot{y},y;x,\theta_{disc}\right)={\left\|D_{e}\left(y;x,\theta_{disc}\right)-D_{e}\left(\dot{y};x,\theta_{disc}\right)\right\|}_{2}$$

(16) $$L_{ad}=E_{x∼P\left(x\right)}\left[\log\left(1-D\left(G\left(x\right)\right)\right)\right]$$

*L*_2_ Loss differentiates the convoluted features between the real and generated images, referred to as perceptual loss. This loss was mostly applied in higher resolution fields to retain the enriched structure-preserving [58].

This section describes different types of loss functions that utilize in lane marking detection applications. There are also many other loss functions available in lane detection applications, though they are combined or part of mentioned loss functions.

#### 2.2.8. Post-Processing

The post-processing step is required if the result from the neural network is the predicted lane coordinates. Clustering or curve-fitting approaches can be applied to transform these points into mathematical descriptions.

DBSCAN has been used mostly to interface the predicted lane pixels with the input images. DBSCAN works more efficiently than other clustering techniques like K-means in arbitrary and noisy clusters [74]. As the lanes’ positions are close to each other and arbitrary, such as straight or curved, DBSCAN would increase efficiency in interfacing the lane pixels. The closest distance point in DBSCAN depends on the value of ε and the minimum number of points for considering the same region. If the lane point is less or equal to the mentioned eps point, it would be considered in the same lane. On the contrary, the point would be considered as in a different cluster. The process would be continued according to the predicted information until all the lanes’ points are converged.

The clustering process makes the lane coordinate into different clusters. It is also challenging to transform different clusters into a mathematical description. As mentioned in the introduction section, distinct types of curve fitting function, such as Catmull–Rom spline, cubic B-spline, and parabolic are used for curve fitting, and cubic B-spline has shown more promising results [23].

#### 2.2.9. Summary of the LMD Network

This section contains Table 1 and Table 2, which summarize different deep learning techniques used in the lane marking detection application and compares the performance of various models. As mentioned earlier, the network summary is also categorized as a single-stage and two-stage architecture with pros and cons.

**Table 1:** Summary of lane detection techniques using DNN.

Author	Deep Learning Technique	Categories	Achievement	Limitation
Single stage				
Li et al. [49]	IANet	Segmentation	Suitable for two-class segmentation	High computation due to non-local features
Gurghian et al. [18]	DeepLane	Classification	Fast detection with simple architecture	Application scenarios are limited
Van et al. [70]	DLFNet	Segmentation	It does not have a predefined condition	Applicable for the fixed number of lanes
Ze et al. [69]	RLaneNet	Regression	Capable of handling uncertain lane numbers without post-processing	The lane ordinate needs to be predefined.
Hou et al. [22]	self-attention distillation	Segmentation	The strategy is more efficient	High computational complexity
Kim et al. [68]	TLELane	Segmentation	Significant achievement on the small dataset	It can only detect the ego lane
Davy et al. [63]	Lanenet	Segmentation	Capable of handling uncertain lane number	High computational complexity due to the H-Net
Xingang et al. [20]	SCNN	Segmentation	Slice convolution for long lane	High computational complexity
Shriyash et al. [32]	CooNet	Regression	Less computational network as does not require clustering	Applicable for the fixed number of lanes
Two-stage				
Ghafoorian et al. [19]	EL-GAN	Segmentation	Can capture lane close to the label	Require a high number of parameters
Qin et al. [61]	CNN-LSTM	Segmentation	Useful for the occlusion scene	Computational is complex
Zhang et al. [59]	GLCNet	Segmentation	Capable of making efficient interlinks between subsections of the network	High computational complexity and difficulties in the training stage
Chen et al. [40]	LMD based on VGG16	Segmentation	Dilated convolution can expand the predicted field	The performance result is lower
Huang et al. [28]	Spatial and temporal-based CNN	Object Detection	Spatial and temporal enrich the detection area	Complex architecture
Seokju et al. [27]	VPGNet	Object Detection	Efficient in different environmental conditions	High computational complexity due to the post-processing
Huval et al. [25]	EELane	Object Detection	Effective for the occlusion scene	It contains the perpetual prediction
Kim et al. [17]	RANSAC	Classification	Overcome the limitations of traditional approaches	The structure of the network is not accurate enough

**Table 2:** Summary of the performances among various deep learning techniques.

Authors	Detection Rate (%)	FPR (%)	FNR (%)	Recall (%)	Accuracy (%)	Precision (%)
Jongin et al. [75]	93	-	-	-	-	-
Dan et al. [76]	-	-	10.03	-	-	-
Soonhong et al. [77]	88.70	-	-	-	-	-
Bei et al. [73]	-	-	-	92.8	-	95.49
Xue et al. [78]	-	5.5	-	-	-	-
Gurghian et al. [18]	-		-	99.9	-	98.96
He et al. [79]	-	-	-	93.80	-	95.49
Kim et al. [80]	98	-	-	-	-	-
Seokju et al. [27]	87	-	-	88	-	-
Zhe et al. [81]	-	2.79	4.99	95.01	-	94.94
Umar et al. [82]	99	-	-	-	-	-
Davy et al. [63]	-	7.8	2.44	-	96.38	-
Ghafoorian et al. [19]		4.12	3.36	-	96.39	-
Xingang et al. [20]	-	6.17	1.8	-	96.53	-
Ze et al. [69]	-	3.9	-	-		-
Youjin et al. [83]	92.4	-	-	-		-
Xiaolong et al. [84]	-	1.41	4.53	-	-	95.65
Wenjie et al. [85]	-	7.7	-	-		-
Tian et al. [86]	-	-	-	66.4		83.5
Huang et al. [28]	-	-	-	96.6	-	97.3
Ye et al. [87]	-	-	5.17	-	-	-
Chao et al. [88]	-	-	-	66	96.26	89
Philion et al. [89]	-	7.2	4.5	-	95.2	-
Azimi et al. [90]	-	-		-	85.95	-
Sun et al. [91]	-	2.0	-		96.4	-
Zhang et al. [92]	95.21	-	-	-	-	-
Zou et al. [61]	-	4.24	1.84	95.8	97.2	85.7
Nguyen et al. [93]	-	-	-	-	98.1	-
Hou et al. [22]	-	6.02	2.05	-	96.64	-
Fabio et al. [37]	-	-	-	-	76.53	-
Lo et al. [46]	-	-	-	-	-	-
Zang et al. [94]	82.44	-	-	-		-
Mamidala et al. [95]	-	-	-	-	96.1	-
Liu et al. [96]	-	-	-	-	97.9	-
Ko et al. [97]	-	2.94	2.63	-	96.7	-

## 3. Comparative Analysis

**Figure 13:**
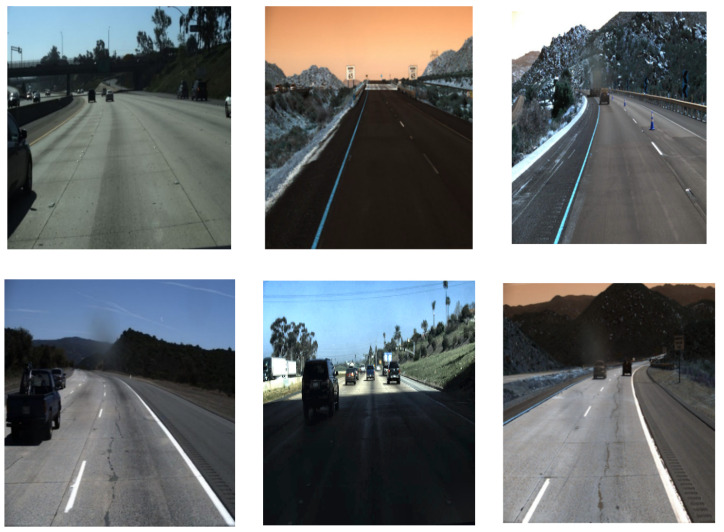
Sample image frames of the Tusimple dataset.

This section describes the constructive comparison of lane marking detection using deep neural networks regarding performance parameters and output visualization. There are five [19,20,61,63,70] segmentation-based DNN which are considered for the comparative sudsy. Moreover, the Tusimple has been considered the largest dataset for lane marking detection since 2018, and is available in [98]. This dataset was also trained and tested on many lane marking detection tasks. Therefore, the Tusimple dataset is considered for performance analysis in this comparative analysis. It contains annotated image frames of different weather conditions, such as straight lane, curve lane, shadow, occluded through vehicles, low light, etc. It has around 3.6k image frames for training and around 2.7k completely unknown image frames for testing. Instead of lane markings, the annotated full lane boundary is the main notability of the Tusimple dataset. The dimension of the images is 720 × 1280. The sample original image frames of the Tusimple dataset have been depicted in Figure 13.

There are particular statistical performance measurement units for evaluating neural network results in the image processing arena. For example, accuracy defines how accurately one model can predict the particular information from the image. Since accuracy cannot only be considered a reliable performance parameter to evaluate the performance that research, the other performance parameters, such as precision, recall, and F1score can make a reliable result to evaluate the framework’s performance. The brief descriptions of the performance parameters are given as follows.

Accuracy is the ratio of the actual true prediction to the total sample data.

$$Accuracy=\frac{Actual\;True\;Prediction}{Total\;Sample\;data}$$

The false-positive score is the ratio of wrongly predicted data to the total number of predicted data.

$$FPS=\frac{False\;Preicted\;Data}{Totla\;Predicted\;Data}$$

The false-negative score is the ratio of missed ground truth data to the total number of ground truth data.

$$FNS=\frac{Missed\;Ground\;truth\;Data}{Totla\;Ground\;truth\;Data}$$

Table 3 illustrates the performance result of different deep neural networks used for lane detection on the Tusimple dataset. All of the networks that are arranged in Table 2 have been discussed in the previous section. The CNN-LSTM technique achieves a higher performance result than other mentioned networks. However, other networks also have a significant role in the lane marking detection application, as they have developed new efficient solutions to perspective research gaps.

**Figure 14:**
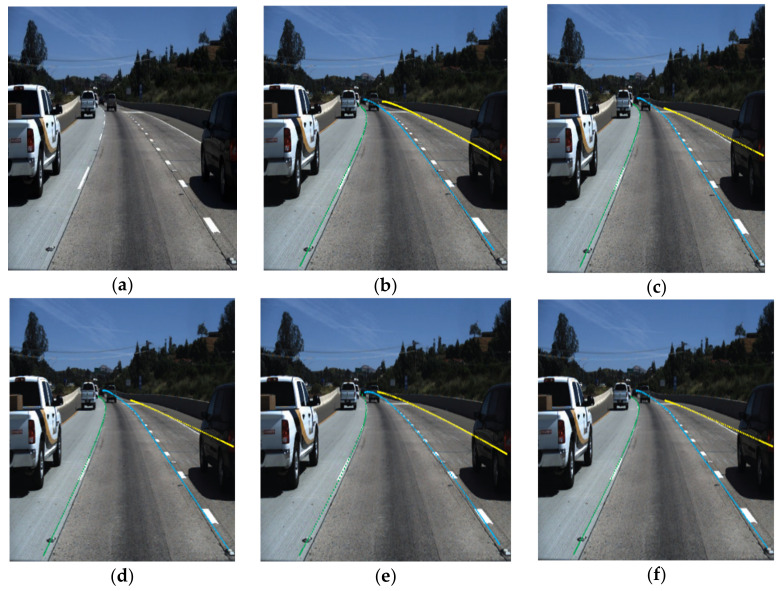
Predicted lane marking using DNN (**a**) Input (**b**) Lanenet (**c**) SCNN (**d**) CNN-LSTM (**e**) ERFNet-DLSF and (**f**) El-GAN.

The final output result of these [19,20,61,63,70] five DNN are depicted in Figure 14. Figure 14 gives a generalized idea about the predicted lane markings from the mentioned networks. A curve lane from the Tusimple dataset is elected for extracting the predicted lane markings.

## 4. Discussion

As lane detection is the ADAS system’s preliminary requirement, it is evident that researchers must develop an advanced model for lane marking detection. This research article discusses a complete overview of lane marking detection systems using deep learning techniques. The main contributions of the article can be categorized into four perspective views. Firstly, it describes different deep learning techniques according to their category in lane marking detection applications so that the researchers can find a specific path to implement neural network techniques in this particular application. Secondly, it describes different loss functions in order to help find a way to improve the performance. Thirdly, it also elaborates on the process of simplification and optimization of the network for simplifying the network architecture. Finally, comparative performance results with visualization of the final output of five existing techniques on the Tusimple dataset are elaborated, which will provide a track to the reader regarding the performance and optimization of the proposed perspective models.

Many vision-based computer-aided features are incorporated into the modern vehicle due to the improvement of the GPU and computational power of the hardware. Though previous researchers have done a tremendous task on lane marking detection, there are still many challenges to address. The first one would be the generalization problem, and better performance can be obtained by transplanting into CNN from the proposed method of [20,22]. Additionally, the supervisor learning process has a deficiency in the appropriate adjustment of the dataset’s different situations. As the neural network utilizes many parameters, real-time application and mobility are also quite challenging for this application.

Some other clues can be followed for promising results focusing on these challenges. As semantic segmentation has computation complexity regarding embedded liability and more efficient accuracy, CNN approaches might be investigated. Again, the supervised learning process can be transformed into a semi-supervised learning approach, as supervisor learning expects a vast amount of annotated data and computational time. Accurate and optimized lane marking detection systems can be designed under different critical situations through meta-learning. Significant feature extractors and detectors may arise from the existing segmentation architecture.

## Figures and Tables

**Table 3:** Summary of lane detection techniques using DNN.

Authors	DNN Method	FPS	FNS	Accuracy
Davy et al. [63]	Lanenet	7.8	2.44	96.38
Ghafoorian et al. [19]	EL-GAN	4.12	3.36	96.39
Xingang et al. [20]	SCNN	6.17	1.8	96.53
Qin et al. [61]	CNN-LSTM	0.01416	0.0186	97.30
Hou et al. [22]	Self-attention distillation	6.02	2.05	96.64
Van et al. [70]	ERFNet-DLSF	0.1064	0.0983	93.38
